# Genome and pan-genome analysis of a new exopolysaccharide-producing bacterium *Pyschrobacillus* sp. isolated from iron ores deposit and insights into iron uptake

**DOI:** 10.3389/fmicb.2024.1440081

**Published:** 2024-08-06

**Authors:** Afef Najjari, Marwa Jabberi, Saïda Fatma Chérif, Ameur Cherif, Hadda Imene Ouzari, Javier A. Linares-Pastén, Haitham Sghaier

**Affiliations:** ^1^Laboratoire de Microbiologie et Biomolécules Actives (LR03ES03), Faculté des Sciences de Tunis, Université Tunis El Manar, Tunis, Tunisia; ^2^Laboratory “Energy and Matter for Development of Nuclear Sciences” (LR16CNSTN02), National Center for Nuclear Sciences and Technology (CNSTN), Sidi Thabet Technopark, Ariana, Tunisia; ^3^ISBST, LR11-ES31 BVBGR, University of Manouba, Biotechpole Sidi Thabet, Ariana, Tunisia; ^4^Biochemistry and Molecular Biology Lab of Faculty of Sciences of Bizerte, Risks Related to Environmental Stress, Struggle and Prevention (UR17ES20), University of Carthage, Bizerte, Tunisia; ^5^Laboratoire de Matériaux, Cristallochimie et Thermodynamique Appliquée, Faculté des Sciences de Tunis, Université de Tunis El Manar, Tunis, Tunisia; ^6^Institut Préparatoire aux Etudes d’Ingénieurs—El Manar, Université de Tunis El Manar, El Manar II, Tunis, Tunisia; ^7^Department of Biotechnology, Faculty of Engineering, Lunds Tekniska Högskola (LTH), Lund University, Lund, Sweden

**Keywords:** heavy metal removal, uptake of siderophores, metal biosorption, ***Psychrobacillus*** new species, ferrienterobactine, ferribacillibactine

## Abstract

Bacterial exopolysaccharides (EPS) have emerged as one of the key players in the field of heavy metal-contaminated environmental bioremediation. This study aimed to characterize and evaluate the metal biosorption potential of EPS produced by a novel *Psychrobacillus* strain, NEAU-3TGS, isolated from an iron ore deposit at Tamra iron mine, northern Tunisia. Genomic and pan-genomic analysis of NEAU-3TGS bacterium with nine validated published *Psychrobacillus* species was also performed. The results showed that the NEAU-3TGS genome (4.48 Mb) had a mean GC content of 36%, 4,243 coding sequences and 14 RNA genes. Phylogenomic analysis and calculation of nucleotide identity (ANI) values (less than 95% for new species with all strains) confirmed that NEAU-3TGS represents a potential new species. Pangenomic analysis revealed that *Psychrobacillus* genomic diversity represents an “open” pangenome model with 33,091 homologous genes, including 65 core, 3,738 shell, and 29,288 cloud genes. Structural EPS characterization by attenuated total reflectance-Fourier transform infrared (ATR-FTIR) spectroscopy showed uronic acid and α-1,4-glycosidic bonds as dominant components of the EPS. X-ray diffraction (XRD) analysis revealed the presence of chitin, chitosan, and calcite CaCO_3_ and confirmed the amorphous nature of the EPS. Heavy metal bioabsorption assessment showed that iron and lead were more adsorbed than copper and cadmium. Notably, the optimum activity was observed at 37°C, pH=7 and after 3 h contact of EPS with each metal. Genomic insights on iron acquisition and metabolism in *Psychrobacillus* sp. NEAU-3TGS suggested that no genes involved in siderophore biosynthesis were found, and only the gene cluster *Feu*ABCD and trilactone hydrolase genes involved in the uptake of siderophores, iron transporter and exporter are present. Molecular modelling and docking of *Feu*A (protein peptidoglycan siderophore-binding protein) and siderophores ferrienterobactine [Fe^+3^ (ENT)]^-3^ and ferribacillibactine [Fe^+3^ (BB)]^-3^ ligand revealed that [Fe^+3^ (ENT)]^-3^ binds to Phe122, Lys127, Ile100, Gln314, Arg215, Arg217, and Gln252. Almost the same for [Fe^+3^ (ENT)]^-3^ in addition to Cys222 and Tyr229, but not Ile100.To the best of our knowledge, this is the first report on the characterization of EPS and the adsorption of heavy metals by *Psychrobacillus* species. The heavy metal removal capabilities may be advantageous for using these organisms in metal remediation.

## 1 Introduction

The genus *Psychrobacillus* belongs to the *Bacillaceae* family within the Firmicutes phylum. It was proposed by Krishnamurthi in 2010 after reclassification of three *Bacillus* strains under the genus *Psychrobacillus* based on polyphasic approaches (Krishnamurthi et al., 2010), including *Bacillus insolitus* DSM 5 T 55, *Bacillus psychrotolerans* DSM 11706 T and *Bacillus psychrodurans* DSM 11713 T; these species were transferred to the new genus *Psychrobacillus* and reclassified. *Psychrobacillus* species are considered psychrophiles, which can grow at 0–20°C, and psychrotolerants (or psychrotrophic), which can grow up to ~30°C (DasSarma and DasSarma, 2018). *Psychrobacillus* harbors Gram-positive, endospore-forming motile rods and aerobic bacteria. At the time of writing, nine validly described species: *Psychrobacillus insolitus* isolated from soil (Krishnamurthi et al., 2010), *Psychrobacillus psychrodurans and Psychrobacillus psychrotolerans* isolated from garden soil (Abd El-Rahman et al., 2002), *Psychrobacillus soli* isolated from oil-contaminated soil (Pham et al., 2015), *Psychrobacillus vulpis* isolated from faeces of a red fox (Rodríguez et al., 2020), *Psychrobacillus lasiicapitis* isolated from the head of an ant (Shen et al., 2017), *Psychrobacillus* isolated from an iceberg in Antarctica (Choi and Lee, 2020), *Psychrobacillus faecigallinarum* isolated from faeces of hen (Gilroy et al., 2021) and *Psychrobacillus antarcticus* isolated from soil sampled at Vale Ulman, King George Island (Antarctica) (Da Silva et al., 2023). Such species may tend to evolve several genes in their genome to cope with various habitats, leading to genome-wide diversity.

Using the pangenome strategy represents an appropriate method to reveal genetic variation, species diversity and evolution that could explain the adaptation of *Psychrobacillus* species to different ecological niches (Hollensteiner et al., 2023). Bacterial pangenomes represent the gene set of all species strains (Medini et al., 2005; Vernikos et al., 2015; Chaudhari et al., 2018). It includes a group of core genes that are common to all strains. The accessory genes include dispensable genes shared by two or more strains. They are crucial for environmental adaptation, genome evolution, and unique genes specific to a single strain (Zakham et al., 2021). In recent years, whole-genome sequencing analysis has provided comprehensive information on inter- and intra-species genome variation through pan-genome analysis (Naithani et al., 2023). Such genetic variation may also be responsible for the open or closed structure of the pangenome (Tettelin et al., 2005), depending on the capacity of the species for the uptake of exogenous DNA, the availability of the machinery for its utilisation and the abundance of ribosomal RNA (Diene et al., 2013). In the first part of this study, we report the in-depth genomic and pan-genomic analysis of a new bacterium, *Psychrobacillus*, isolated from an ores deposit sampled from the Tamra iron mine (Nefza mining district, N. Tunisia).

As reported in previous studies, most *Psychrobacillus* species were isolated from extreme environments, and to cope with those conditions, they may employ several strategies, one of which is to produce extracellular polymeric substances (EPSs), which act as a barrier to protect the cell from radiation (Guesmi et al., 2019), desiccation, freezing environments (Corsaro et al., 2004; Carrión et al., 2015), heavy metals (Guibaud et al., 2003; Gupta and Diwan, 2017; Cao et al., 2023), etc. Microbial EPSs are a group of high molecular weight biopolymers produced during the metabolic process that can be attached to the cell surface or released into the environment (Nwodo et al., 2012). They are reported to play an important role in bioremediation processes due to their structural and functional properties (Ates, 2015), especially for removing heavy metals, as well as emulsifying, bio-flocculating, and antioxidant agents (Ahmad et al., 2015; Chouchane et al., 2020; Da Silva et al., 2022). Several studies have been carried out on EPSs produced by different bacterial species; however, no studies have been conducted on EPS from *Psychrobacillus* species. In the second part of our study, we focused on the production, structural characterisation, and determination of metal bioabsorption by the EPS produced by the isolated *Pyschrobacillus* bacteria.

Given the origin of isolation (iron ores deposit), the third part of our study focuses on analysing the iron uptake and metabolism systems of the *Pyschrobacillus* bacterium based on whole genome mining. Iron is a crucial element for most living organisms. It is essential for many biological functions such as DNA synthesis and repair, genomic stability, respiration, and photosynthesis, and as a cofactor in many biochemical reactions (Andrews et al., 2003; Liu et al., 2022). Although it is abundant on Earth, the bioavailability of iron is minimal. To remedy this, microorganisms have developed siderophore-dependent iron uptake strategies designed to solubilise and capture iron in aerobic environments (Hoegy et al., 2005). Siderophores are low-molecular-weight (less than 10 kDa) molecules with a high affinity for ferric iron (Fe^3+^) (Khan et al., 2018). They are generally classified based on the coordination groups that chelate the Fe^3+^. The most common groups are catecholates (phenolates), hydroxamate siderophores and carboxylate siderophores (Saha et al., 2016). The import of siderophore-Fe^3+^ complexes is internalised by siderophore-binding proteins located on the bacterial surface. In Gram-positive bacteria, these proteins include ABC transporters with an ATP cassette located on the cytoplasmic membrane (Braun and Hantke, 2011), the case of *B. subtilis*, which employs bacillibactin as an endogenous siderophore and may subsequently import exogenous siderophores like enterobactin, petrobactin or hydroxamate siderophores like ferrichrome (Miethke et al., 2013). It is worth noting that various known siderophores can present significant structural homologies, enabling bacteria to use other siderophores produced by different bacteria (xenosiderophores) (Will et al., 2023). Several studies have reported the iron acquisition system within Gram-positive bacteria; however, no studies have been conducted on *Psychrobacillus* species. Here, we sought to analyse the insights into iron uptake and metabolism of the new *Psychrobacillus* sp. bacterium NEAU-3TGS isolated from an iron ores deposit from the Tamra iron mine (Nefza mining district, Northern Tunisia).

## 2 Materials and methods

### 2.1 Strain isolation and molecular identification

The bacterial strain NEAU-3TGS was isolated from an iron ores deposit collected from northern Tunisia’s Tamra iron mine (Nefza mining district) (Longitude: 9°06.7226′E, Latitude: 37°02.7413′N). The mine has been exploited for about a century and belongs to the Nappe Zone of northern Tunisia. At the time of sampling, the temperature was 8°C, a pH of 6.85. The mineralogical characterisation of the iron ores deposit is shown in Supplementary Table S1.

Isolation was performed on tryptic soy agar (TSA) medium (Sigma Aldrich) using the serial dilution technique. The iron ore sample was first ground, and then 1 g was added to 9 mL of physiological saline water (0.9% NaCl), and then a serial dilution was performed on tryptic soy agar (TSA) medium (Sigma Aldrich). Plates were incubated at 28°C for 48 h. Identification of the NEAU-3TGS strain was based on partial 16S rRNA gene sequencing. DNA extraction was performed using sodium dodecyl sulphate proteinase K treatment (Cherif et al., 2003). 16S rRNA gene amplification was performed using the universal bacterial primers 27F (5’-TCC GGT TGA TCC TGC RG-3′) and 1492R (5′-GGT TAC CTT GTT ACG ACT T3-’) (El Hidri et al., 2013). The PCR reaction mixture, containing PCR buffer (1X), MgCl2 (1.5 mM), 0.25 mM of each dNTP, 0.5 μM of each primer, 0.1 μg of chromosomal DNA, and 1 U of Taq DNA polymerase (Fermentas), was used to in 50 μL to perform PCR reactions programmed as follows: 95°C for 5 min; 35 cycles of [94°C 45 s, 55°C 45 s and 72°C 1 min], and a final extension step at 72°C for 10 min. PCR products were Sanger sequenced with an automated capillary ABI Biosystem 3,130 (MrDNA). The 16S rRNA gene sequence obtained was compared to sequences deposited on the EzBioCloud server (Yoon et al., 2017). The sequence for the 16S rRNA gene was deposited in GenBank under the accession number OR577132.

### 2.2 Genome assembly, annotation, and phylogenomic analysis of NEAU-3TGS strain

Genome sequencing and assembly were performed using Ion Torrent sequencing technology. Sequencing quality control of raw sequence data was performed using the FastQC algorithm (version 0.11.8),^1^ and poor-quality reads were trimmed using the Trimmomatic tool (Bolger et al., 2014). *De novo* assembly using the SPAdes tool (version 3.13.1) (Bankevich et al., 2012). Then, the quality of the assembled genome was assessed using QUAST software (Meier-Kolthoff Gurevich et al., 2013). To determine the relationship of NEAU-3TGS with *Psychrobacillus* species, we first perform the whole-genome-based taxonomic analysis through the Type Strain Genome Server (Meier-Kolthoff and Göker, 2019). Pairwise genomic comparisons were calculated, and intergenomic distances were inferred using the algorithm “trimming” and distance formula d5 using 100 distance replicates in FastME 2.0 (Lefort et al., 2015). The genome of strain NEAU-3TGS was annotated using several tools. The first was the rapid annotation subsystem technology (RAST) server (Aziz et al., 2008). Then, the predicted gene sequences were translated and searched in the National Center for Biotechnology Information (NCBI) non-redundant database and the Kyoto Encyclopedia of Genes and Genomes (KEGG) database with a cutoff value of 0.01 (Kanehisa and Goto, 2000). Indeed, ortholog prediction and functional genes annotation, including clusters of orthologous genes (COGs), were identified using EGGNOG 5.0 database and OrthoFinder software (Huerta-Cepas et al., 2019). We used FastTree with the maximum likelihood (Felsenstein, 1981) method to construct phylogenetic trees based on conserved single-copy gene sequences. The whole Genome Shotgun project has been deposited at DDBJ/ENA/GenBank under the accession JAPNOZ000000000. The version described in this paper is version JAPNOZ010000000.

### 2.3 Comparative genome analysis and pangenome inference of *Psychrobacillus* strains

Here, we used one of the representative genome sequences of nine *Psychrobacillus* species that have been validated in the LPSN database (Parte et al., 2020) and whose genome sequences are available in GenBank^2^ (as of May 2024). We first checked the similarity of the NEAU-3TGS genome sequence with all species by comparing values of average nucleotide identity analysis (ANI), calculated using an OrthoANI in OAT (the Orthologous Average Nucleotide Identity Tool) (Lee et al., 2016). The species includes *Psychrobacillus faecigallinarum* (NZ_JACSQO000000000.1), *Psychrobacillus glaciei* (NZ_CP031223.1), *Psychrobacillus insolitus* (NZ_QKZI00000000.1), *Psychrobacillus lasiicapitis* VDGH01 (NZ_VDGH00000000.1), *Psychrobacillus psychrodurans* DSM30747 (NZ_JAMKBI000000000.1), *Psychrobacillus psychrotolerans* (NZ_FOXU00000000.1), *Psychrobacillus soli* (NZ_VDGG00000000.1), *Psychrobacillus vulpis* (NZ_VDGI00000000.1) and *Psychrobacillus antarcticus* (JAIZDB000000000.1). To assess the genetic and phylogenetic diversities of *Psychrobacillus* species, we performed a pangenome analysis using the Roary version 3.13.0 pipeline (Page et al., 2015) with an identity threshold of 90% BLASTp percentage. Roary output files were used to analyse and visualise the primary and accessory genomes of *Psychrobacillus* strains using R.^3^

### 2.4 Extraction of EPS

A qualitative assessment of EPS production was performed on a TSA medium supplemented with 2% glucose, and mucoid colonies characterised the EPS phenotype. The extraction of EPS was performed according to the protocol described by Guesmi et al. (2022). Briefly, pre-grown strains were inoculated into 200 mL of tryptic soy broth (TSB) medium (Sigma Aldrich) supplemented with 2% glucose and incubated at 30°C for 72 h. The culture was then centrifuged at 16,000 g for 20 min, and the supernatant was added with 3 volumes of absolute ethanol and incubated at 4°C for 24 h. Precipitated EPS were collected by centrifugation at 16,000 g for 20 min, and the pellet was dried at room temperature for 24 h. The dried pellet was dissolved in distilled water to remove proteins from crude EPS, treated with 4% (w/v) trichloroacetic acid (TCA) for 6 h and centrifuged. The supernatant EPS fraction was again purified with ethanol and left overnight at 4°C, and after centrifugation, the EPS was dried at room temperature until it reached constant weight (Shameer, 2016).

The protein content was analysed using the Bradford method (Bradford, 1976) with bovine serum albumin (BSA) as a standard. The absorbance was read at 595 nm. The content of polynucleotides (DNA and RNA) was estimated by measurement of optical density (OD) at 260 and 280 nm using a NanoDrop 2000c spectrophotometer (Thermo Fisher Scientific Inc., United States).

### 2.5 EPS analysis

Dried EPS samples were analysed by attenuated total reflectance-Fourier transform infrared spectroscopy (ATR-FTIR) and X-ray diffraction. ATRS-FTIR spectra of EPS were recorded in the transmission mode, using a Mattson FTIR spectrophotometer in the range of 400 to 4,000 cm^−1^, where the functional groups of EPS were identified. The X-ray study was conducted using a D8 ADVANCE Bruker diffractometer with CuK_α_ radiation (*λ* = 1.5418 Å) in the 2θ range of 5–80°. The 2θ values of diffraction peaks were plotted, and the distances (*d*) between two lattice planes were determined using Bragg’s law (Eq. 1):


(1) $$d=\frac{\lambda }{2\sin\left(\theta \right)}$$

Where *λ* represents the wavelength of the incident X-rays, *d* is the spacing between crystal lattice planes, and θ is the X-ray beam’s incidence angle.

The Crystallinity Index (CI) was computed with the formula (Eq. 2):


(2) $$CI=\frac{\mathrm{Peak}\;\mathrm{areas}\;\mathrm{of}\;\mathrm{crystals}}{\mathrm{Peak}\;\mathrm{areas}\;\mathrm{of}\;\mathrm{crystals}+\mathrm{Peak}\;\mathrm{area}\;\mathrm{of}\;\mathrm{amorphous}\;\mathrm{peak}}\;\times 100$$

### 2.6 Metal bioabsorption assessment of EPS

The evaluation of the metal biosorption potential of EPS (1.0 mg/100 mL) against the heavy metals, iron (FeCl_3_), lead (PbCl_2_), copper (CuSO_4_) and cadmium (CdCl_2_) at 1000 ppm for each metal salt based on the method of Shameer (2016) with some modifications including the contact time (1, 2, 3, 6, and 24 h) at pH 7 and 37°C, the pH (5, 6, 7, and 8) at 37°C for 24 h and the temperature (20, 28, 37, and 45°C) at pH 7 for 24 h. After the adsorption process, the solution was centrifuged at 24,000 g for 10 min to separate the EPS. All assays were performed in triplicate. The metal biosorption was measured by atomic absorption spectroscopy based on the following calculation (Shameer, 2016):

Metal removal (*q*) from solution expressed as mg metal removed/g dry weight ^(−1)^ (Volesky and Holan, 1995):


$$q\;\left(\mathrm{mg}\;g^{-1}\right)=V\;\left(\mathrm{Ci}-Cf\right)\;m^{-1}\text{.}$$

Where V: sample volume (l); Ci: initial concentration, Cf: final concentration; m: the amount of dry biomass (g).

### 2.7 Molecular modelling

The molecular structure of the protein FeuA was obtained through the deep learning program RoseTTAFold (Baek et al., 2021). FeuA was subjected to docking with the ligands [Fe+3(ENT)]^−3^ and [Fe^+3^(BB)]^−3^. The atomic coordinates of the ligands were obtained from the crystal structures available in the Protein Data Bank (PDB) 2XUZ and 2WHY, respectively. Dockings were performed with AutoDock Vina (Trott and Olson, 2010), considering the total flexibility of the ligands.

## 3 Results and discussions

### 3.1 Isolation and comparative genomic analysis of the NEAU-3TGS strain

**Figure 1:**
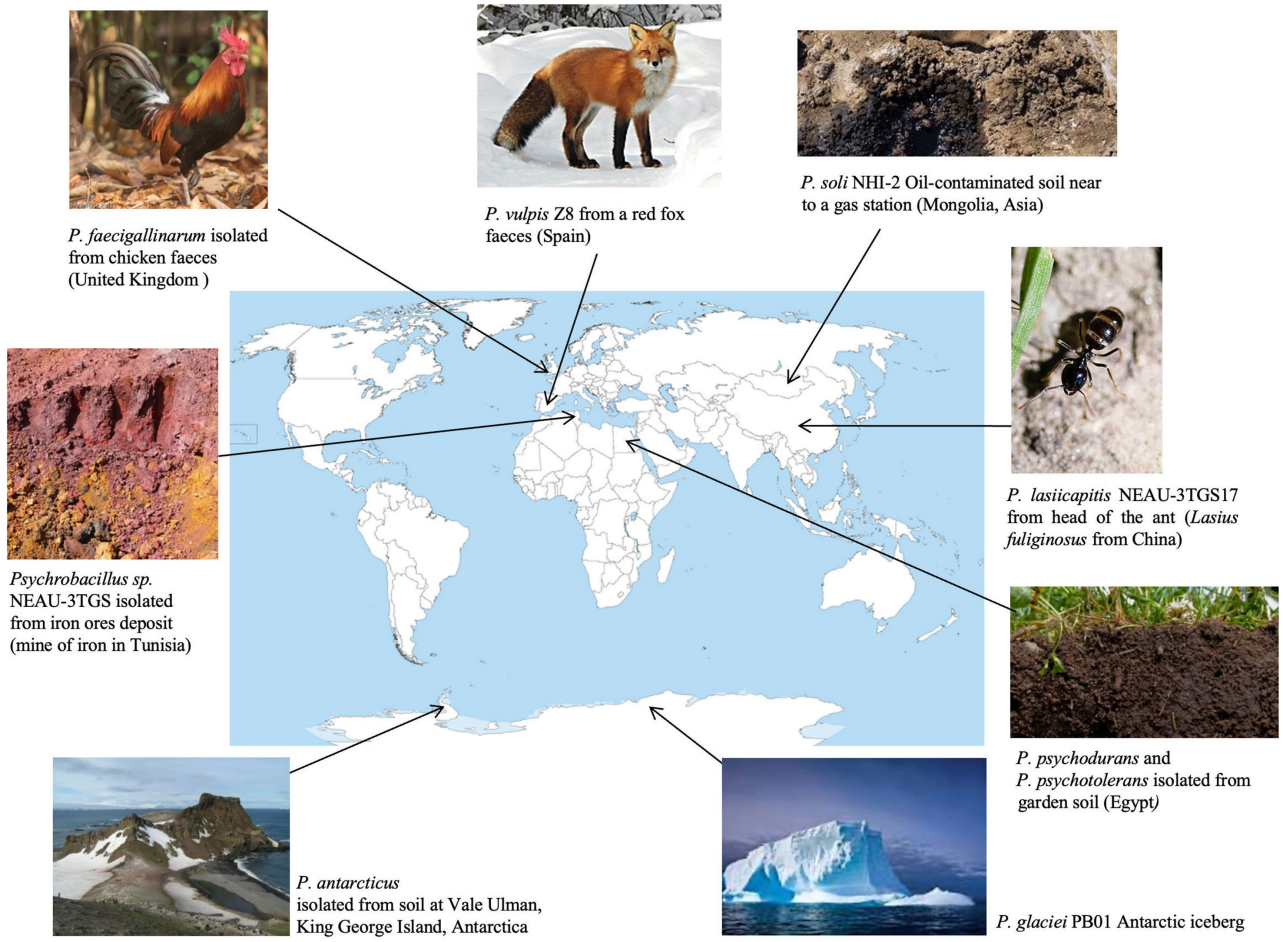
Sources of isolation of *Psychrobacillus* sp. NEAU-3TGS (this study) and the 9 validated *Psychrobacillus* species available in LPSN as of May 2024 (Parte et al., 2020).

NEAU-3TGS bacteria showed white colonies of rod-shaped Gram-positive cells (Supplementary Figure S1). The NEAU-3TGS isolate identification based on partial 16S rDNA gene sequencing (570 bp) (GeneBank: OR577132) gave 99.65% of identity to the species *P. lasiicapitis* (GeneBank: KP219721). *Psychrobacillus* is a member of the *Bacillaceae* family within the Firmicutes phylum. This genus was proposed by Krishnamurthi in 2010 after re-examining certain *Bacillus* species, whereby *Bacillus insolitus*, *Bacillus psychrotolerant* and *Bacillus psychrodurans* were reclassified within the new genus *Psychrobacillus* (Krishnamurthi et al., 2010), which encompasses *P. lasiicapitis*, *P. psychrodurans*, *P. psychrotolerans*, *P. soli*, *P. insolitus*, *P. glaciei*, *P. faecigallinarum*, *P. vulpis* and *P. antarcticus* (Parte et al., 2020) (Table 1). *Psychrobacillus* are renowned for their ability to thrive at cold temperatures. They have been identified and isolated from cold environments, such as polar icebergs, and other habitats like garden soils, petroleum-contaminated soils, and red fox faeces (Choi et al., 2020) (Figure 1).

**Table 1:** Genomic features of *Psychrobacillus* species.

Species	Accession number	No of scaffolds	Length (MB)	GC%	Proteins	rRNA operon copy number	Completeness (%)	ANI *vs* NEAU-3TGS strain (%)	Origin of isolation
*Psychrobacillus* sp. NEAU-3TGS	JAPNOZ000000000	16	4.41	37.00	4,243	14	99.34	–	Iron (This study)
*P. lasiicapitis* NEAU-3TGS17	VDGH01000000	28	4.48	37.06	4,294	19	100	93.47	Head of the ant (*Lasius fuliginosus*, China) (Shen et al., 2017)
*P. soli* NHI-2	VDGG00000000	101	4.22	37.12	3,993	22	100	85.01	Oil-contaminated soil near a gas station in Mongolia, Asia (Pham et al., 2015)
*P. vulpis* Z8	VDGI00000000	76	4.02	35.89	3,807	23	99.34	78.78	Faeces of a red fox from Spain (Rodríguez et al., 2020)
*P. glaciei* PB01	CP031223	2	4.35	35.97	3,952	33	99.34	77.91	Antarctic iceberg (Choi and Lee, 2020)
*P. psychrotolerans* DSM 11706	FOXU00000000	20	3.60	36.36	3,549	23	99.34	75.89	Soil from Egypt (Abd El-Rahman et al., 2002; Krishnamurthi et al., 2010)
*P. insolitus* DSM 5	QKZI00000000	33	3.28	36.02	3,200	26	99.68	76.63	NA
*P. faecigallinarum* Sa2BUA9	JACSQO000000000	5	4.00	36.48	3,855	32	100	74.22	Faeces from *Gallus gallus* from United Kingdom (Gilroy et al., 2021)
*P. psychrodurans* DSM 11713	JAMKBK000000000	48	4.02	36.00	3,844	45	99.34	76.17	Soil from Egypt (Abd El-Rahman et al., 2002; Krishnamurthi et al., 2010) (Egypt)
*P. antarcticus* val9	JAIZDB000000000.1	87	3.98	36.60	3,799	18	99.14	76.97	Isolated from the soil at Vale Ulman, King George Island, Antarctica (Da Silva et al., 2023)

The average nucleotide identity (ANI) was measured versus the chromosome of *Psychrobacillus* sp. NEAU-3TGS strain.

**Figure 2:**
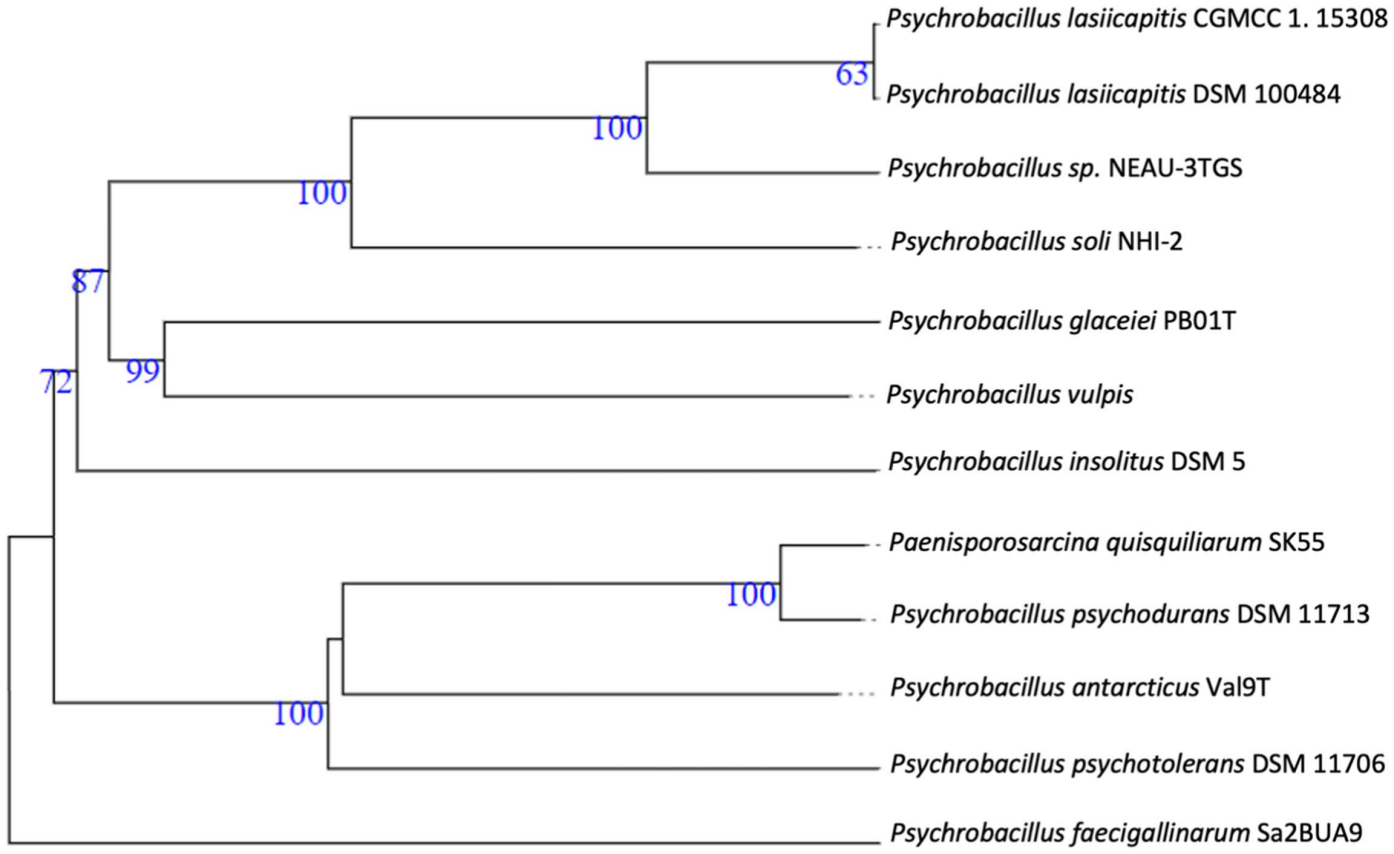
Whole-genome-based phylogenetic tree highlighting the position of *Psychrobacillus* NEAU-3TGS strain to other closely related bacterial taxa. Trees are generated with Fastme 2.1.6.1. The numbers are above 60% from 100 replications. The tree was rooted at the midpoint.

The whole assembled genome of the NEAU-3TGS strain contains 4.418 Mb in length, consisting of 16 scaffolds, with an N50 of 666.8 kb and a G + C content of 37.0% (Table 1). Phylogenomic analysis and genome-based taxonomical classification of the NEAU-3TGS strain showed that the NEAU-3TGS strain is identified as a potential new species and was placed with the same clade of *P. lasiicapitis* species represented by the two strains CGMCC 1.15308 and DSM 100484 (Shen et al., 2017) (Figure 2). To confirm whether *Psychrobacillus* sp. NEAU-3TGS is a novel species. ANI analysis was carried out with the genome sequences of representative strains belonging to the eight LPSN-validated *Psychrobacillus* species (Table 1), including *P. lasiicapitis* NEAU-3TGS17 (VDGH01000000) isolated from ant head (Shen et al., 2017), *P. glaciei* PB01 (NZ_CP031223.1) isolated from an Antarctic iceberg (Choi and Lee, 2020), *P. soli* NHI-2 (NZ_VDGG00000000.1) isolated from contaminated soil (Pham et al., 2015), *P. psychrodurans* (NZ_JAMKBI000000000.1) and *P. insolitus* DSM 5 (QKZI00000000) isolated from garden soil (Abd El-Rahman et al., 2002; Krishnamurthi et al., 2010), *P. psychrotolerans* DSM 11706 (FOXU00000000) isolated from soil (Abd El-Rahman et al., 2002), *P. faecigallinarum* (NZ_JACSQO000000000.1) isolated from chicken feed (Gilroy et al., 2021), *P. vulpis* Z8 (NZ_VDGI00000000.1) isolated from red fox feed (Rodríguez et al., 2020) and *P. antarcticus* Val9 (JAIZDB000000000.1) isolated from soil (Da Silva et al., 2023). Results revealed that ANI values ranged between 74.22 and 93.47% for *P. faecigallinarum* (JACSQO000000000.1) and *P. lasiicapitis* NEAU-3TGS17 respectively, which were below the threshold value (95%) for distinguishing the different species (Goris et al., 2007) (Table 1). Accordingly, the ANI indicates that strain NEAU-3TGS represents a novel species in the *Psychrobacillus* genus.

Comparison of general genomic characteristics of *Psychrobacillus* species showed similar profiles except for chromosome size, varying from 3.28 Mb to 4.48 Mb for *P. insolitus* and *P. lasiicapitis* NEAU-3TGS17, respectively, and for rRNA operon copy numbers which range from 14 in *Psychrobacillus* sp. NEAU-3TGS to 45 in *P. psychrodurans* DSM 11713 (Table 1). This may reflect the environmental adaptation of *Psychrobacillus* species. In general, the ribosomal RNA operon represents a fundamental genetic structure in protein synthesis and, thus, a functional trait linked to the bacterial life cycle (Klappenbach et al., 2000; Stevenson and Schmidt, 2004), while bacteria with a higher number of rRNA copies can better withstand nutrient fluctuations and tend to inhabit nutrient-poor environments (Klappenbach, 2001; Jeyasingh and Weider, 2007).

Genome annotation of *Psychrobacillus* sp. NEAU-3TGS based on the RAST server revealed 4,746 DNA coding sequences (CDS) distributed in 417 subsystems with uneven distribution (Supplementary Table S2). The subsystems with the highest presence of CDS were (i) amino acids and their derivatives (535 CDS); (ii) carbohydrates (370 CDS); (iii) protein metabolism (233 CDS); (iv) cofactors, vitamins and prosthetic groups (201 CDS); (v)RNA metabolism (186); (vi) membrane transport (180 CDS); (vii) fatty acids, lipids and isoprenoids (173 CDS); (viii) DNA Metabolism (124 CDS); and (ix) cell wall and capsule (124) (Supplementary Table S2). In addition, the genome of this strain presented CDS potentially resistant toxic compounds (46 CDS), including genes implicated in copper homeostasis (*n* = 4), cobalt-zinc-cadmium resistance (*n* = 10), zinc resistance (*n* = 3), arsenic resistance (*n* = 8), cadmium resistance (*n* = 1) and resistance to chromium compounds (*n* = 1) (Supplementary Table S3). These gene fears could be correlated to the origin of strain, the contamination of iron mines with heavy metals (Decrée et al., 2008). RAST annotation was conducted for the other species, and results showed almost the same distribution of subsystem feature counts except for phages and prophages, which were absent for *P. psychotolerans* and *P. psychrodurans* (Supplementary Table S2). In addition, COG annotation of the CDSs of *Psychrobacillus* sp. NEAU-3TGS genome sequence revealed 23 COGs where the majority were assigned to unknown function (S, 22.97%), followed by amino acid transport and metabolism (E, 9.54%), translation, ribosomal structure, and biogenesis (J, 4.94%), inorganic ion transport and metabolism (P, 4.77%), and energy production and conversion (C, 4.31%). The remaining clusters are below 4%. Compared to other species, almost the same profile has been observed (Supplementary Table S4).

### 3.2 Pan-genome analysis of *Psychrobacillus* species

**Figure 3:**
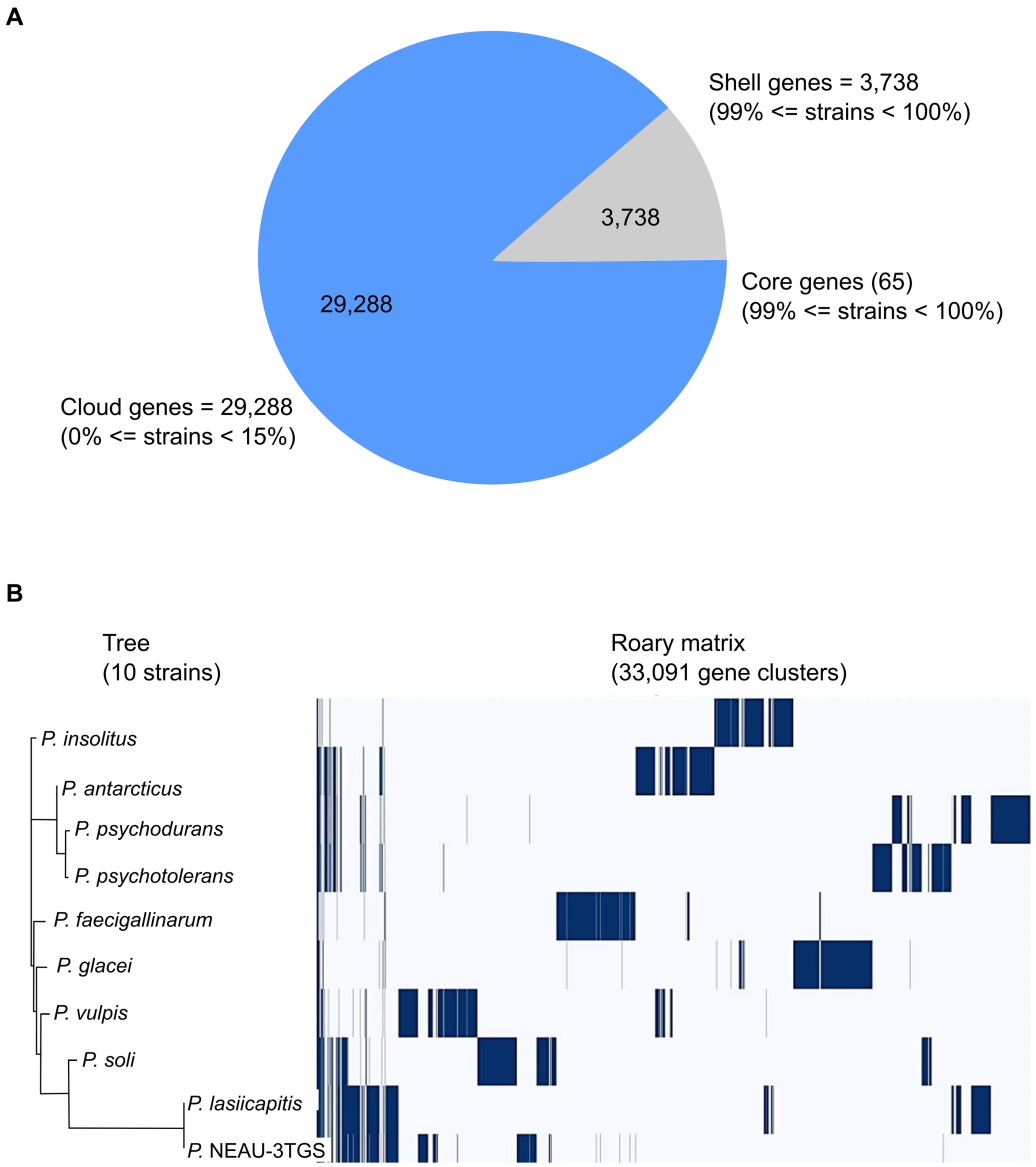
Pangenome analysis of ten strains of *Psychrobacillus* species by Roary. **(A)** Pie chart representation of pangenome of *Psychrobacillus* strains. The chart shows the gene distribution in the pangenome [Core genes (99% < strains <= 100%), shell genes (15% <= strains <= 95%) and cloud genes (0% <= strains <= 95%)]. **(B)** Phylogenetic tree of gene presence/absence matrix from pangenome analysis. Blue: the presence of the gene. White: the absence of the gene.

The genomic diversity of *Psychrobacillus* species undertaken in this study was performed based on pangenome analysis using the Roary (version 3.13.0) pipeline with a 90% identity threshold of BLASTp percentage. The results showed that the pangenomic structure of the ten *Psychrobacillus* strains comprises a total of 33,091 homologous genes where (i) 65 are core genes (99% <= strains <= 100%), (ii) 33,026 are accessory genes present in <95% genomes composed of 3,738 shell genes (15% <= strains <95%) and 29,288 cloud genes (0% <= strains <15%). No soft genes (95% <= strains <99%) were identified (Figure 3A). The heatmaps (Figure 3B) generated based on the matrix of the presence or absence of genes (Supplementary Table S5) in *Psychrobacillus* species pangenomes display the variation in the distribution of accessory genes (Figure 3B). In general, pangenome gene families harbour the genetic determinants of species; core genome genes are typically involved in bacterial replication, translation, and maintenance of cellular homeostasis (Carlos Guimaraes et al., 2015; Brockhurst et al., 2019), and accessory genes are involved in responding to environmental changes during adaptive evolution (Brockhurst et al., 2019). Notably, we identified a high degree of heterogeneity among *Psychrobacillus* species, with many cloud genes (single genes) versus core genes (conserved genes). These single genes were unevenly distributed among species, with the lowest numbers in *P. lasiicapitis* (*n* = 1,546) versus *Psychrobacillus* sp. NEAU-3TGS (*n* = 1,654), *P. psychotolerans* (*n* = 2,622), *P. antarcticus* (*n* = 2,915), *P. soli* (*n* = 3,082), *P. soli* (*n* = 3,082), *P. insolutis* (*n* = 3,096), *P. psychodurans* (*n* = 3,270), *P. vilpus* (3,480) and *P. faecigallinarum* (*n* = 3,789) (Figure 2B and Supplementary Table S5). This analysis is consistent with a previous study on five *Psychrobacillus* species (Choi et al., 2020).

**Figure 4:**
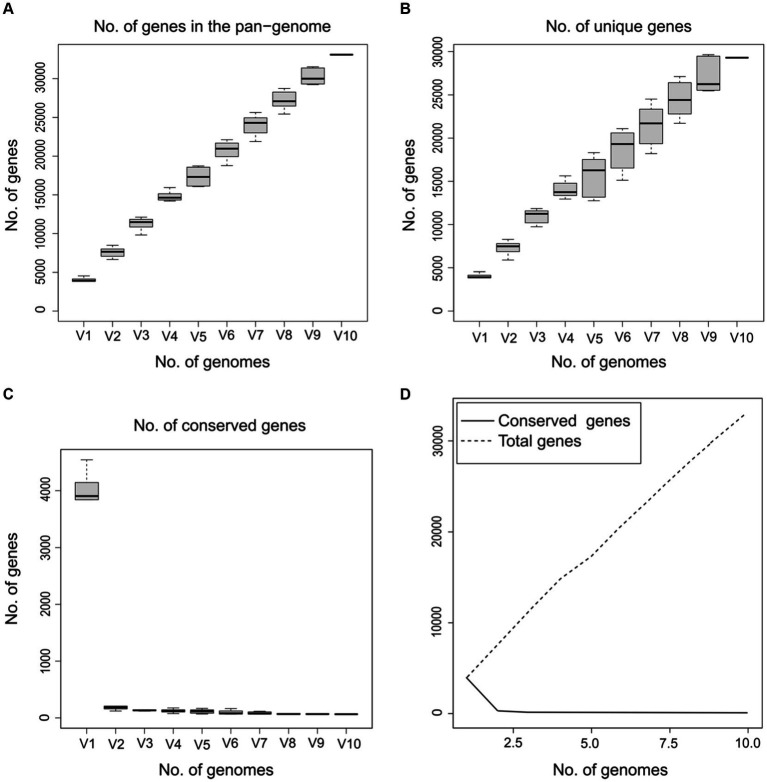
Gene discovery graphs for the ten *Psychrobacillus* strains demonstrating that as the total number of genes increases (V1–V10), the number of total **(A)** and unique genes increase **(B)** and the number of core genes gradually decreases **(C,D)**.

On the other hand, to determine whether the pangenome of *Psychrobacillus* strains is open or closed, the number of core and pangenome genes was plotted as a function of the number of cumulative genomes (V1–V10). The results showed that the size of the pangenome and the number of new genes increase steadily with the addition of each further genome (V1–V10), unlike the conserved genes, where they remain stable with low numbers (Figure 4). Accordingly, *Psychrobacillus species* had a large open pan-genome. Generally, members of species with open pan-genomes have relatively small core genomes with a more significant percentage of accessory genes within each genome, which is consistent with our results (Donati et al., 2010; Whitworth et al., 2021). Open pan-genomes profile of *Psychrobacillus* species may be correlated to the source of isolation and its geographical location, which always adds up new genes by horizontal gene transfer (HGT); typically, species living in different habitats benefit from various microbes a genetic material exchange and therefore continue to expand their total number of genes (Brockhurst et al., 2019; Chhotaray et al., 2020). *Psychrobacillus* species investigated in this analysis have been isolated from various ecological habitats, citing soils from Egypt, Gallus faeces from the UK, red fox faeces from Spain, an iceberg and soil from Antarctica, and head from China, oil-saturated soil and ferrous metal (Figure 1).

### 3.3 Production and structural property studies of exopolysaccharide and heavy metal bioabsorption evaluation

*Psychrobacillus* sp. NEAU-3TGS colonies exhibited a mucoid morphology on TSA medium supplemented with 2% glucose (Supplementary Figure S1), indicating extracellular sucrase activity (Malik et al., 2009). Genome mining for genes implicated in EPS production based on RAST annotation showed that *Psychrobacillus* sp. NEAU-3TGS contains 4 genes, including *eps*B (Manganese-dependent protein-tyrosine phosphatase), gene involved in the regulation of EPS biosynthesis (ii) *eps*C (Tyrosine-protein kinase transmembrane modulator) and *eps*D (Tyrosine-protein kinase), genes implicated on chain length determination of EPS (iii) *eps*E (Undecaprenyl-phosphate galactosephosphotransferase, gene involved on the biosynthesis of the repeating sugar units), followed by GT1 (glycosyltransferase de group 1) GT2 (glycosyltransferase de group 2) genes implicated in the process of adding glycosyl groups to the growing EPS chain. Glycosyltransferases (GTs) play a critical role in the biosynthesis of exopolysaccharides. They catalyse the formation of glycosidic linkages between sugar residues (Wang et al., 2020). EPS gene clusters vary among bacteria and are often called the *eps*ABCDE stretch. They are typically conserved and present in a specific order and play critical roles in the biosynthesis of exopolysaccharides (Deo et al., 2019).

NEAU-3TGS EPS was isolated and purified to a total dry weight of 20.5 mg per 100 mL and used for structural characterisation by ATR-FTIR and DRX analysis.

#### 3.3.1 Powder X-ray diffraction analysis

**Figure 5:**
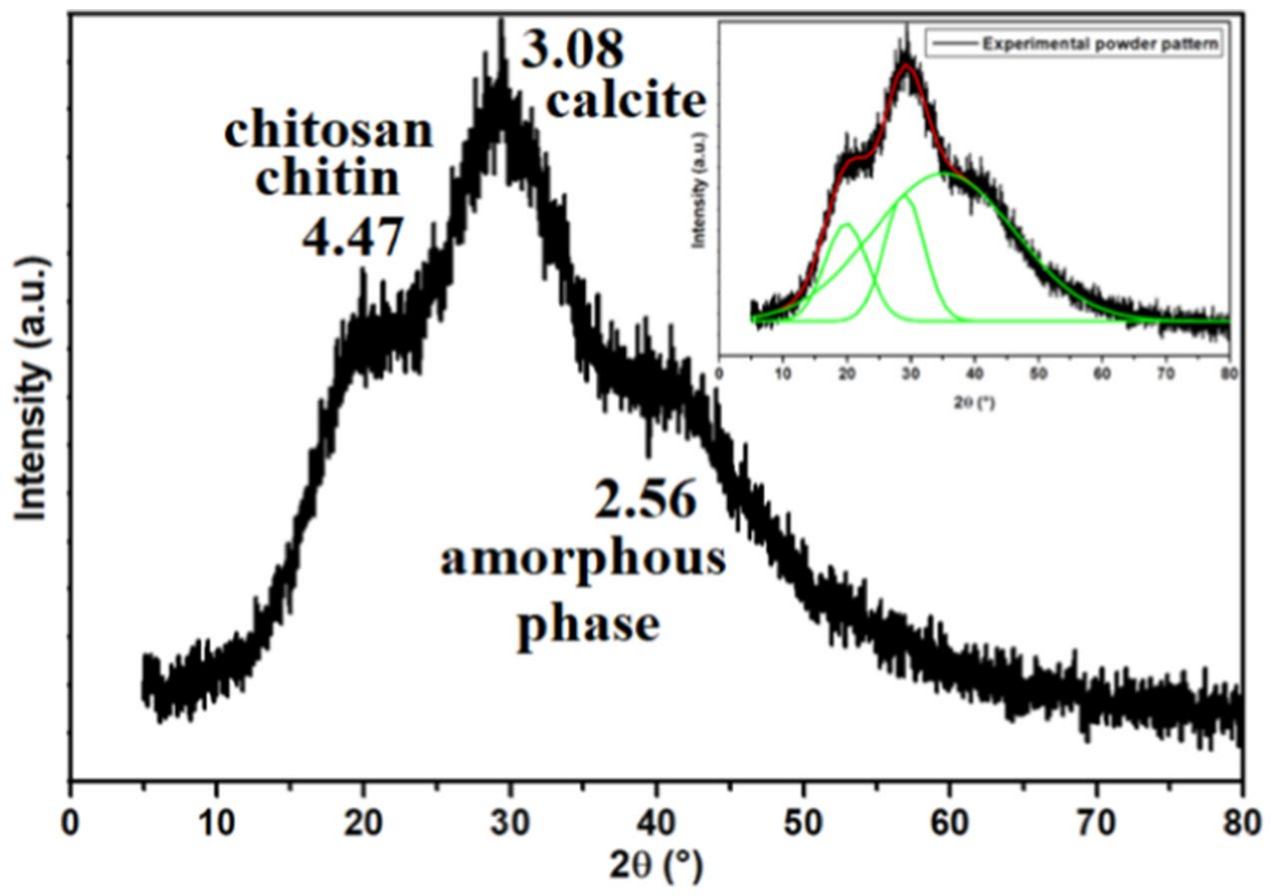
X-ray powder pattern of the exopolysaccharide. The inset illustrates the Gaussian fit result.

Figure 5 displays the X-ray powder pattern of EPS, revealing three peaks around 15–40° (2θ). These peaks suggest the semi-crystalline and amorphous nature of EPS (Dogan et al., 2015; Mosharaf et al., 2018; Solmaz et al., 2018; Krishnamurthy et al., 2020; Mathivanan et al., 2021; Nwaiwu et al., 2021; Vinothkanna et al., 2022). For all the peaks observed in the XRD powder pattern, we have calculated the distance (*d*) and the Crystallinity Index (CI), as listed in Table 2.

**Table 2:** The distance (*d*) and the crystallinity index (CI) of all peaks observed in the XRD powder pattern.

Peaks	Distance *d* (Å)	Crystallinity index CI (%)
Peak 1	4.47	13.69
Peak 2	3.08	16.70
Peak 3	2.56	69.61

Nicolaus et al. (2002) reported that EPS produced by thermophiles marine strains contains 81% carbohydrate, 3% protein, and 2% nucleic acid. The same study determined that the EPS was composed of glucose, mannose, galactose, and mannose-amine monomers by GC–MS analysis. Additionally, Dogan et al. (2015) reported that EPS produced by thermal *Bacillus* contains 80% of amorphous phases, including the presence of chitin, chitosan, protein, and calcite. EPS’s Crystallinity Index (CI) value was determined to be 20%, indicating a semi-crystalline nature and the predominance of the amorphous phase. Based on Figure 5 and Table 2 and Dogan et al.’s findings (Dogan et al., 2015), the presence of chitin and chitosan is inferred, both observed at 4.47 Å with a CI of 13.69%. The presence of calcite (CaCO_3_) is suggested at a distance of 3.08 Å, with a CI of 16.70%. An amorphous phase is indicated at 2.56 Å, with a dominant CI of 69.61%. Solmaz et al. (2018) also observed the presence of chitin and/or chitosan.

Several studies have shown that some composites of EPS, such as chitin and chitosan, have a potential for bioremediation (Samoila et al., 2019). For instance, they can be used to discolour textile dyes and remove ionic metals from wastewater (Fatima, 2021).

#### 3.3.2 Attenuated total reflectance-Fourier transform infrared spectroscopy studies

**Figure 6:**
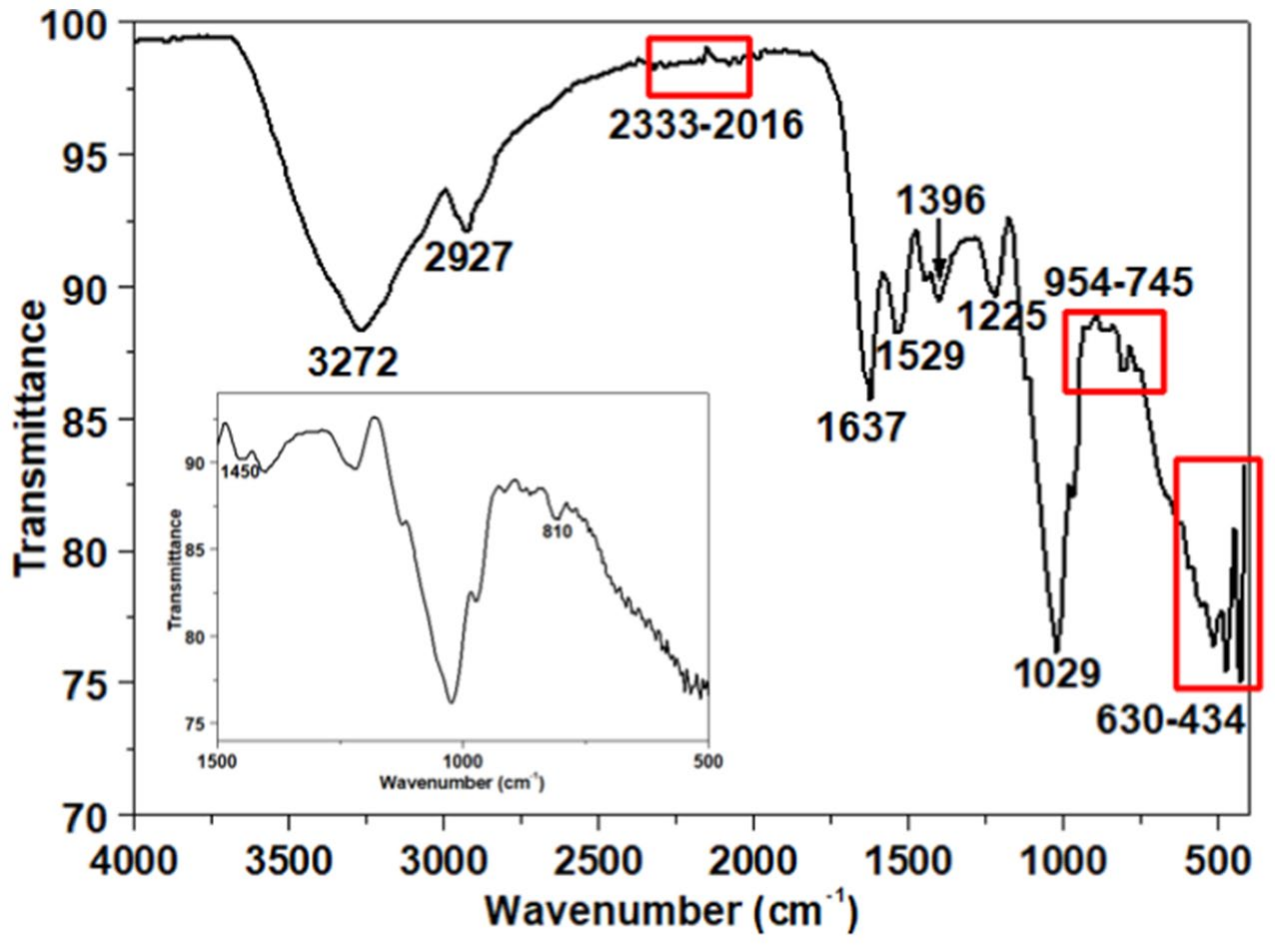
ATR-FTIR spectrum of the exopolysaccharide. The inset illustrates the spectrum from 1,500 to 500 cm^−1^.

ATR-FTIR analysis spectrum is depicted in Figure 6. The assignment of peaks to specific functional groups was based on previous studies (Badireddy et al., 2010; Jiao et al., 2010; Brian-Jaisson et al., 2016; Wang et al., 2016; Maddela et al., 2018; Hong et al., 2021; Wang et al., 2022). The peak at 3272 cm^−1^ is indicative of the stretching vibration associated with both hydroxyl groups (−OH) of carbohydrates in polysaccharides and amino groups (−NH_2_ or −NH) of proteins (Braissant et al., 2007; Tapia et al., 2009; Liang et al., 2010; Deng et al., 2022; Wang et al., 2022). The absorption peak at 2927 cm^−1^ (Braissant et al., 2007) corresponds to the C–H stretching vibration of aliphatic −CH_2_ or −CH_3_ groups, revealing the carbohydrate structure of EPS (Parikh and Madamwar, 2006) and indicating the presence of sugar content (Iyer et al., 2005). The IR peaks observed in the range of 2,333–2016 cm^−1^ may be attributed to CO_2_ adsorption or the asymmetric stretching of −N = C = O− groups (Mishra and Jha, 2009).

Furthermore, the ATR-FTIR spectrum shows two peaks at 1637 cm^−1^ and 1,529 cm^−1^, attributed to amides I and II of the secondary protein structure within the EPS (Maruyama, 2001; Badireddy et al., 2008, 2010; Sheng et al., 2013). The symmetric stretching of C=O in the—COO^−^ is represented by a peak at 1396 cm^−1^ (Nara et al., 1994; Omoike and Chorover, 2004; Pongjanyakul and Puttipipatkhachorn, 2007; Badireddy et al., 2008; Freitas et al., 2009; Sheng et al., 2013). A minor peak at 1225 cm^−1^, associated with N − H bending and C − N stretching vibrations (Coates, 2000), was also observed. A peak at 1029 cm^−1^ indicates the presence of uronic acid’s o-acetyl ester linkage bonds (Bramhachari and Dubey, 2006). Additionally, the 954–745 cm^−1^ range confirms the presence of an α-1,4-glycosidic bond (Maddela et al., 2018). Firm absorption peaks in the 630–434 cm^−1^ range are attributed to the deformation of—COO^−^ groups in polysaccharides (Wiercigroch et al., 2017).

Based on DRX analysis, chitin, chitosan, calcite (CaCO_3_) and an amorphous phase were identified. The IR spectrum analysis supports these findings, revealing characteristics peaks indicative of chitosan and chitin (notably at 3272, 1637, 1,529, 1,396, and 1,225 cm^−1^), corresponding to N-H stretching of amine groups, C=O stretching related to the amide I band, and N-H bending associated with the amide II band (Dahmane et al., 2014; Negrea et al., 2015). For the calcite phase, characteristic peaks at 1450 and 810 cm^−1^ were observed (inset Figure 6), corresponding to the asymmetric stretching and bending vibrations of the carbonate ion (CO_3_^2−^) present in calcium carbonate (Singh and Sawant, 2022).

Overall, the FTIR spectra decipher the functional groups of the carbohydrate polymer, which are reported to play an essential role in the functional and biological activities of the EPS. For example, the presence of hydroxyl (OH) and aliphatic (CH_2_) groups makes the EPS either hydrophilic or hydrophobic, making it an appropriate emulsifying agent (Sathiyanarayanan et al., 2016). Indeed, the presence of carboxylate (COO^−^) functional groups allows these polymers to bind to other oppositely charged molecules, such as heavy metals (Donot et al., 2012).

**Figure 7:**
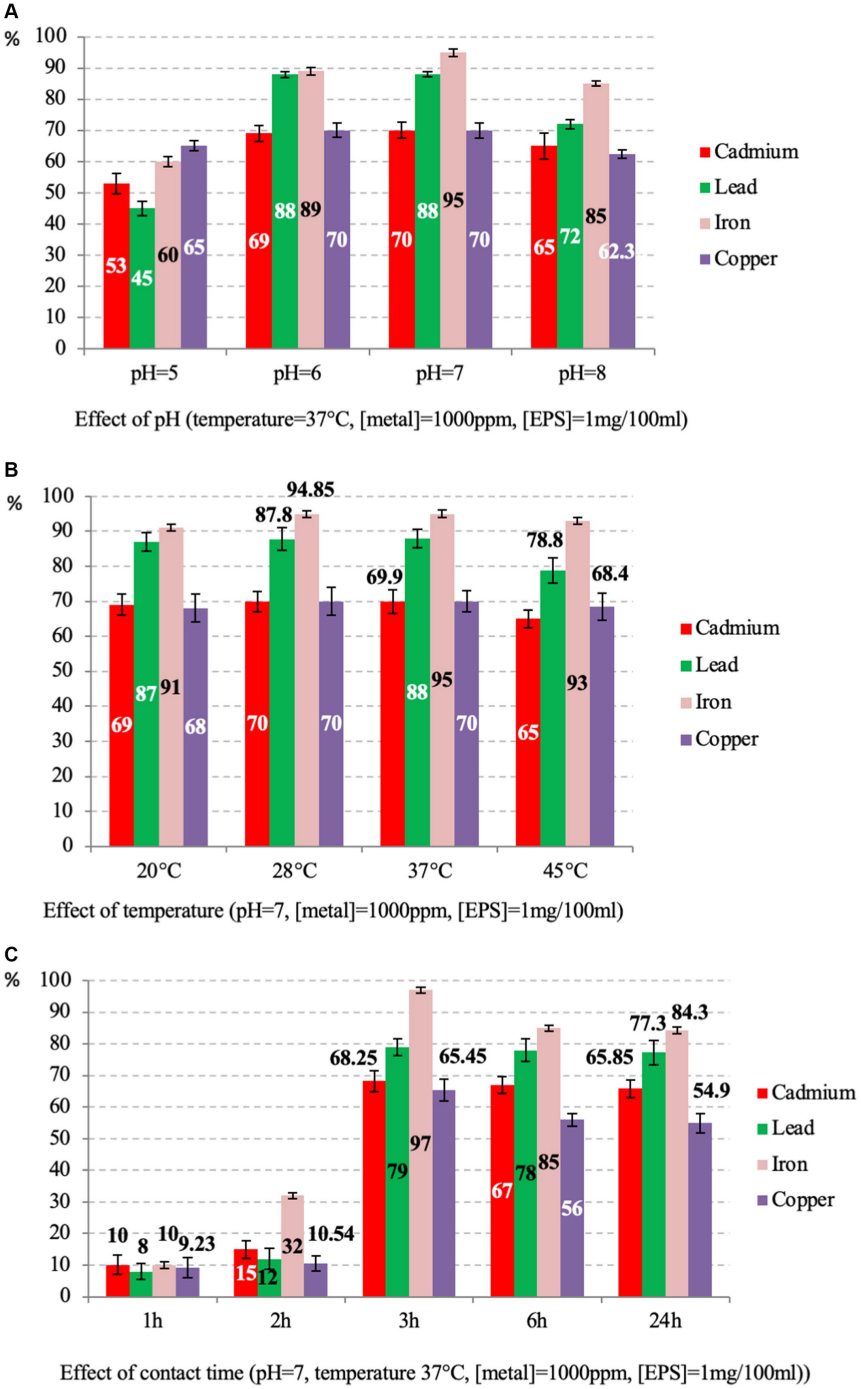
Percentage of EPS adsorption rates of caddmium, lead, iron and copper at various parameters, including pH **(A)**, temperature **(B)** and contact time **(C)**.

Based on the structural properties of *Psychrobacillus* sp. NEAU-3TGS EPS’, we have assessed its ability to remove iron, copper, lead and cadmium ions, with high toxicity and among the priority metals of public health significance (Tchounwou et al., 2012). The adsorption rate of each metal (cadmium, lead, iron, and copper) was determined as a function of contact time, pH, and temperature at 1000 ppm of metal and 1 mg/100 mL of EPS (Figure 7).

The effect of pH was tested at 37°C for 24 h. Results showed that adsorption is moderately affected by pH for all metals (Figure 7A). The maximum activities are observed at pH = 7 with 95, 88, 70, and 70% for iron, lead, cadmium and copper, respectively (*p*-value >0.05 for three replicates per test). These results are consistent with those reported on the EPS of *Bacillus*-related members conducted under the same conditions for three isolates, citing *Bacillus licheniformis*, *Bacillus cereus* and *Bacillus subtilis*, where lead was the most adsorbed compared to copper and cadmium (Shameer, 2016; Biswas et al., 2020; Krishnamurthy et al., 2020; Zainab et al., 2020). The different removal efficiency observed for iron (Fe^3+^) compared to lead (Pb^2+^), copper (Cu^2+^), and cadmium (Cd^2+^) could be attributed to the different charge density of the ions, which depends on the ionic size of the cations (Salehizadeh and Shojaosadati, 2003). Other studies have reported that the rate of adsorption of metal ions by the EPS of some *Bacillus*-related members varies depending on bacteria species, metal and pH of the solution. For instance, for *Bacillus mucilaginosus*, the iron adsorption was optimal at pH 5.0 (Yang et al., 2017). For *Bacillus firmus*, the optimal pH for lead, copper, and zinc was 4.5, 4, and 6, respectively (Salehizadeh and Shojaosadati, 2003). Differences in removal efficiency could also be attributed to the different charge densities of the ions, which depend on the cations’ ionic size and the solution’s pH (Salehizadeh and Shojaosadati, 2003). The pH of the solution significantly affects the adsorption process of metal ions. At low pH values, the high concentration of H^+^ ions competes with metal ions for adsorption sites and at high pH values, the negative charge density on the EPS surface may increase due to deprotonation of metal binding sites, thus decreasing metal adsorption (Kim et al., 1996; Can and Jianlong, 2007; Wei et al., 2016).

The influence of temperature on the adsorption rate was tested at pH = 7 for 24 h. The results showed that the values are almost similar, and the best were observed at 37°C with 95, 88, 70 and 69.6% for iron, lead, cadmium and copper, respectively (*p*-value <0.05 for three replicates per test) (Figure 7B). Generally, the effect of temperature on metal adsorption depends on the tested metal and biomolecules, citing the example of *B. cereus*, where the optimal temperature for manganese biosorption was 35°C (Zhenggang et al., 2018).

The contact time was tested at 37°C and pH = 7. The results showed that the uptake rate increased with the time of incubation. The best rate was observed at 3 h for all metals with 97, 79, 68.25, and 65.45% (*p*-value >0.05 for three replicates per test) for iron, lead, cadmium and copper respectively, and a slightly decreasing was observed up to 24 h (Figure 7C). As the contact time increases, the adsorption rate decreases because the metal ions gradually occupy the binding sites, thus reducing the concentration of lead and cadmium ions in the solution (El-Sayed, 2013).

It is worth noting that iron was the most adsorbed in all conditions. These results agree with those reported on the EPS of *Bacillus*-related members (Krishnamurthy et al., 2020; Zainab et al., 2020). In general, EPSs are implicated in treating metal-contaminated environments (Zheng et al., 2008; Chouchane et al., 2020; Siddharth et al., 2021). It is well known that EPSs have a large surface area for interaction, especially with metals, which is helpful for survival in various contaminated areas (Pal and Paul, 2008).

### 3.4 Genomic insights on iron acquisition and metabolism in *Psychrobacillus* sp. NEAU-3TGS

Given its origin of isolation, we were interested in identifying the mechanisms of iron metabolism and uptake used by *Psychrobacillus* sp. NEAU-3TGS, based on RAST annotation. Results revealed that genes encoding for membrane proteins are involved in the uptake of siderophores, iron transporter, and exporter proteins, although no genes involved in siderophore biosynthesis were found. The genes are as follows: iron ABC transporter permease (*n* = 9), iron export ABC transporter permease subunit FetB (*n* = 1), iron-hydroxamate ABC transporter substrate-binding protein (*n* = 1), iron chelate uptake ABC transporter family permease subunit (*n* = 1) and iron-siderophore ABC transporter substrate-binding protein (*n* = 2) (Supplementary Table S6 and Supplementary Figure S2A). Analysis of the iron-siderophore uptake systems revealed that the NEAU-3TGS strain imports both bacillibactin and petrobactin ferric complexes. Siderophore bacillibactin (or corynebactin) is a catechol trilactone secreted by various types of bacilli, notably *Bacillus anthracis* and *Bacillus subtilis* (May et al., 2001; Cendrowski et al., 2004). The compound is mainly utilised to chelate Fe^3+^ present outside the cell and transfer them into the cytoplasm via an ABC2 transporter (Hotta et al., 2010). Ferri-bacillibactin uptake is mediated by the FeuABC transporter and tri-lactone hydrolase (YuiI). FeuA is a periplasmic binding protein component acting as a peptidoglycan siderophore-binding protein. FeuB is a membrane permease component which forms a channel through the bacteria’s inner membrane where iron ions can pass. FeuC is the inner membrane’s ATP-binding cassette (ABC) protein component. It is responsible for binding and hydrolysing ATP, providing the energy necessary to transport iron ions through the membrane. FeuB interacts with FeuC and works in concert with the uptake of iron ions into the bacterial cell. FeuD is an ATPase component of an ABC-type iron-compound transporter. Iron is liberated from the Fe-bacillibactin complex upon transport through hydrolysis mediated via the trilactone hydrolase (YuiI), which can cleave the siderophore. The gene cluster is organized in an apparent single operon under Fur family transcriptional regulator (Fur) containing FeuABCD and Fur regulation gene (Supplementary Figure S2B).The gene cluster FeuABCD and trilactone hydrolase (BesA) was identified in *Bacillus*-related members such as *B. anthracis, B. subtilis*, *B. cereus*, *B. thuringiensis* and *B. subtilis* groups (Miethke et al., 2006; Abergel et al., 2008; Hayrapetyan et al., 2016). Furthermore, other studies reported that the FeuABC-YusV transporter of *B. subtilis* was shown to import ferric complexes of both bacillibactin and enterobactin siderophores (Miethke et al., 2006).

Concerning the siderophore petrobactin (anthrachelin), the uptake within NEAU-3TGS strain is mediated by Fe-ABC1 (Iron compound ABC uptake transporter substrate-binding protein), the Fe-ABC2 (Iron compound ABC uptake transporter permease protein) and Fe-ABC3 (Iron compound ABC uptake transporter ATP-binding protein) (Supplementary Figure S2C). Siderophores petrobactin is a highly specific iron (III) transport ligand (Rue and Bruland, 1995; Manck et al., 2022). The iron-chelated petrobactin complex is easily subjected to photolytic oxidative decarboxylation due to its α-hydroxy carboxylate group, converting iron (III) to the more biologically useful iron (II) (Barbeau et al., 2002). Previous studies have elucidated the petrobactin-iron complex receptor (FhuA), import permeases (FpuB/FatC/FatD), ATPases (FpuC/FatE), and the petrobactin exporter (ApeX) (Hagan et al., 2018). Genome mining for iron metabolism was also assessed for the nine *Psychrobacillus* species, and the results showed that it is like the NEAU-3TGS strain, with no genes implicated in the biosynthesis of siderophore. Indeed, the siderophores petrobactin uptake system was present in all species. However, the bacillibactin uptake system (FeuABCD/YuiI) was present only within the *P. facigallinarum* genome sequence (Supplementary Table S6).

### 3.5 Structural analysis

**Figure 8:**
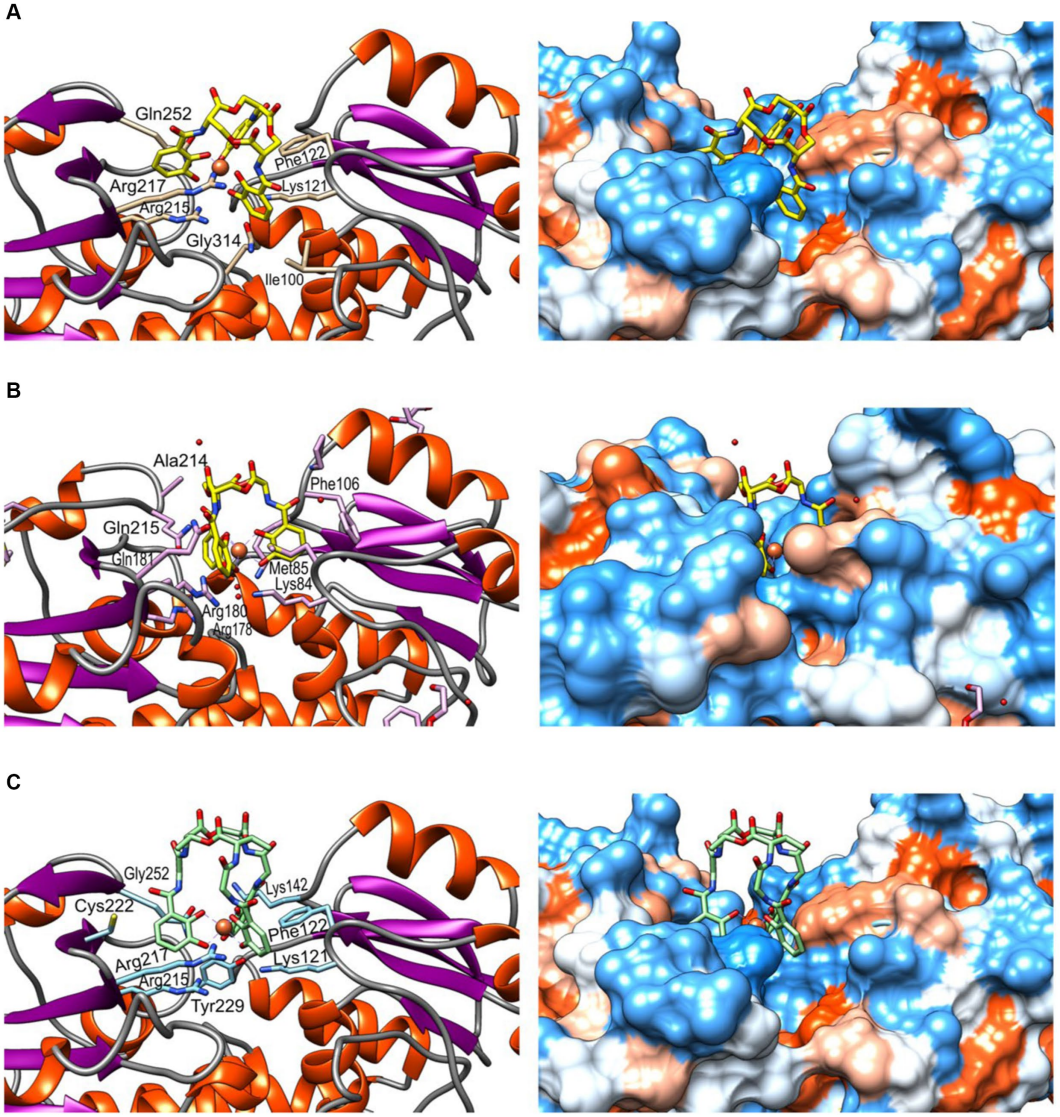
Molecular modelling and docking of FeuA. **(A)** Complex FuA-[Fe^+3^(ENT)]^−3^, **(B)** Complex FuA-[Fe^+3^(ENT)]^−3^ from *Bacillus subtilis*, crystall structure (PDB: 2XUZ) (Peuckert et al., 2011). **(C)** Complex FuA-[Fe^+3^(BB)]^−3^. According to the Kyte-Doolittle scale, the corresponding hydrophobicity surfaces are represented on the left side (Kyte and Doolittle, 1982), ranging from dodger blue for the most hydrophilic to white and orange-red for the most hydrophobic.

The molecular model of FeuA was obtained through deep learning modelling and was used for docking siderophores ferrienterobactine [Fe^+3^(ENT)]^−3^ and ferribacillibactine [Fe^+3^(BB)]^−3^. Gram-negative bacteria, such as *E. coli*, mainly produce enterobactine (ENT). At the same time, becillibactine (BB) is secreted by gram-positive bacteria, such as some *Bacillus* species (*B. antrhacis*, *B. cereus*, *B. subtilis*). Both ENT and BB have binding to the siderophore binding site of FeuA from *Pyschrobacillus* sp. (Figure 8). [Fe^+3^(ENT)]^−3^ binds to Phe122, Lys127, Ile100, Gln314, Arg215, Arg217, and Gln252 (Figure 8A). Compared to the crystal structure of the complex FuA-[Fe^+3^(ENT)]^−3^ from *Bacillus subtilis* (Figure 8B), some conserved amino acids are present: Phe106, Lys84, Arg178, Arg180, and Gln215. In the complex of FeuA from *Pyschrobacillus* sp. and [Fe^+3^(BB)]^−3^ (Figure 8C), most of the same amino acids involved in the interaction with [Fe^+3^(ENT)]^−3^ participate. In addition, Cys222 and Tyr229, but not Ile100. Perhaps the amino acids in the binding site are flexible enough to accommodate slightly different siderophores.

## 4 Conclusion

A new *Psychrobacillus* sp. NEAU-3TGS bacterium was isolated from an iron ore deposit. Pangenome analysis of the nine valid species revealed that *Psychrobacillus* genomic diversity represents an “open” pangenome model. NEAU-3TGS strain showed the potential to produce EPS encoding by *eps*B (manganese-dependent protein-tyrosine phosphatase), *eps*C (tyrosine-protein kinase transmembrane modulator), *eps*D (tyrosine-protein kinase), *eps*E (undecaprenyl-phosphate galactose phosphotransferase) and GT1, 2 (glycosyltransferase de group 1 and 2). Structural characterisation of EPS with XRD analysis showed the presence of chitin, chitosan, and calcite CaCO_3_ and confirmed the amorphous nature of the EPS. On the other hand, ATR-FTIR spectroscopy demonstrated that secondary proteins, uronic acid, α-1,4-glycosidic, and polysaccharides are the main components of the EPS. Heavy metal bioabsorption assessment showed that EPS adsorbs iron, lead, copper and cadmium. Based on the present results, *Psychrobacillus* sp. NEAU-3TGS may be involved in an ecological alternative for heavy metal remediation. *In silico* genomic insights into iron uptake and metabolism showed that only gene cluster *Feu*BCD and trilactone hydrolase gene involved in the uptake of siderophores, iron transporter, and exporter are present in the genome. Molecular modelling and docking of FeuA (protein peptidoglycan siderophore-binding protein) and siderophores ferrienterobactine [Fe^+3^(ENT)]^−3^ and ferribacillibactine [Fe^+3^(BB)]^−3^ ligand revealed that [Fe^+3^(ENT)]^−3^ binds to Phe122, Lys127, Ile100, Gln314, Arg215, Arg217, and Gln252. Almost the same for [Fe^+3^(ENT)]^−3^ in addition to Cys222 and Tyr229, but not Ile100. Further studies could be undertaken to understand the iron metabolism pathway via transcriptomic or proteomic analyses.

## Data availability statement

The datasets presented in this study can be found in online repositories. The names of the repository/repositories and accession number(s) can be found in the article/Supplementary material.

## Author contributions

AN: Conceptualization, Data curation, Investigation, Visualization, Writing – original draft, Writing – review & editing. MJ: Investigation, Methodology, Writing – review & editing. SC: Conceptualization, Data curation, Investigation, Methodology, Writing – original draft, Writing – review & editing. AC: Formal analysis, Resources, Supervision, Writing – review & editing. HO: Formal analysis, Resources, Supervision, Validation, Writing – review & editing. JAL-P: Conceptualization, Formal analysis, Investigation, Methodology, Resources, Software, Supervision, Visualization, Writing – review & editing. HS: Conceptualization, Formal analysis, Resources, Supervision, Validation, Writing – review & editing.

## Footnotes

^1^ http://www.bioinformatics.babraham.ac.uk/projects/fastqc/

^2^ https://www.ncbi.nlm.nih.gov

^3^ https://www.r-project.org/
