# Supplementary material for: Genome and pan-genome analysis of a new exopolysaccharide-producing bacterium Pyschrobacillus sp. isolated from iron ores deposit and insights into iron uptake
Source: Front Microbiol. 2024 Aug 6;15:1440081. doi: 10.3389/fmicb.2024.1440081 (PMC11376405; doi:10.3389/fmicb.2024.1440081)
Supplement: Supplementary file 1 [file Table_1.DOCX]

| Composition | Percentage (%) |
| --- | --- |
| Fe_2_O_3_ | 89.83 |
| SiO_2_ | 15.46 |
| Al_2_O_3_ | 5.74 |
| CaCO_3_ | 4.27% |
| CaO | 0.24 |
| K_2_O | 0.52 |
| MnO | <0.01 |
| ZnO | <0.01 |
| MgO | <0.01 |
| Na_2_O | 0.52 |

**Table S1** : mineralogical characterization of iron ores deposit
