# Supplementary material for: Genome and pan-genome analysis of a new exopolysaccharide-producing bacterium Pyschrobacillus sp. isolated from iron ores deposit and insights into iron uptake
Source: Front Microbiol. 2024 Aug 6;15:1440081. doi: 10.3389/fmicb.2024.1440081 (PMC11376405; doi:10.3389/fmicb.2024.1440081)
Supplement: Supplementary file 2 [file Table_2.DOCX]

**Table S2**: The complete list of the RAST annotated genes of *Psychrobacillus* species.

| **Subsystem Feature Counts** | ***P. vulpis*** | ***P. faecigallinarum*** | ***P. psychotolerans*** | ***P. psychrodurans*** | ***P. insolutis*** | ***P. glaciei*** | ***P. lasiicapitis* NEAU-3TGS17** | ***Psychrobacillus sp.***  ***NEAU-3TGS*** | ***P. soli*** | ***P. antarcticus* val9** |
| --- | --- | --- | --- | --- | --- | --- | --- | --- | --- | --- |
| \| Cofactors, Vitamins, Prosthetic Groups, Pigments \| \| --- \| | 169 | 156 | 159 | 172 | 194 | 150 | 188 | 201 | 217 | 108 |
| Cell Wall and Capsule | 101 | 106 | 113 | 91 | 92 | 136 | 98 | 124 | 98 | 33 |
| Virulence, Disease and Defense | 72 | 61 | 59 | 65 | 53 | 56 | 73 | 73 | 76 | 39 |
| Potassium metabolism | 10 | 9 | 5 | 7 | 5 | 4 | 15 | 7 | 6 | 2 |
| Photosynthesis | 0 | 0 | 0 | 0 | 0 | 0 | 0 | 0 | 0 | 0 |
| Miscellaneous | 36 | 46 | 45 | 45 | 25 | 45 | 49 | 51 | 50 | 24 |
| Phages, Prophages, Transposable elements, Plasmids | 10 | 18 | 0 | 0 | 12 | 8 | 16 | 6 | 3 | 4 |
| Membrane Transport | 114 | 122 | 93 | 98 | 79 | 121 | 167 | 180 | 204 | 45 |
| Iron acquisition and metabolism | 19 | 22 | 18 | 16 | 42 | 20 | 4 | 10 | 4 | 23 |
| RNA Metabolism | 166 | 144 | 149 | 151 | 149 | 184 | 167 | 186 | 163 | 56 |
| Nucleosides and Nucleotides | 115 | 122 | 108 | 115 | 100 | 122 | 142 | 134 | 128 | 106 |
| Protein Metabolism | 208 | 172 | 169 | 238 | 175 | 232 | 245 | 233 | 226 | 145 |
| Cell Division and Cell Cycle | 50 | 48 | 46 | 47 | 50 | 55 | 50 | 52 | 47 | 3 |
| Motility and Chemotaxis | 45 | 47 | 88 | 88 | 38 | 88 | 87 | 91 | 83 | 7 |
| Regulation and Cell signaling | 58 | 50 | 49 | 53 | 47 | 65 | 65 | 73 | 62 | 15 |
| Secondary metabolism | 4 | 4 | 4 | 4 | 4 | 4 | 6 | 4 | 4 | 4 |
| DNA Metabolism | 117 | 112 | 115 | 114 | 112 | 113 | 134 | 124 | 103 | 60 |
| Fatty Acids, Lipids, and Isoprenoids | 133 | 165 | 127 | 139 | 138 | 122 | 178 | 173 | 214 | 47 |
| Nitrogen Metabolism | 7 | 9 | 12 | 22 | 15 | 11 | 12 | 11 | 11 | 7 |
| Dormancy and Sporulation | 16 | 20 | 21 | 23 | 27 | 23 | 8 | 8 | 20 | 7 |
| Respiration | 52 | 61 | 47 | 53 | 51 | 52 | 55 | 55 | 58 | 39 |
| Stress Response | 77 | 86 | 104 | 101 | 86 | 93 | 102 | 117 | 107 | 33 |
| Metabolism of Aromatic Compounds | 34 | 21 | 14 | 14 | 41 | 19 | 28 | 31 | 25 | 6 |
| Amino Acids and Derivatives | 403 | 388 | 352 | 363 | 343 | 398 | 490 | 535 | 471 | 256 |
| Sulfur Metabolism | 14 | 22 | 15 | 18 | 35 | 13 | 24 | 26 | 25 | 9 |
| Phosphorus Metabolism | 54 | 52 | 55 | 59 | 55 | 46 | 63 | 65 | 59 | 15 |
| Carbohydrates | 269 | 299 | 300 | 335 | 233 | 248 | 376 | 370 | 414 | 161 |
