# Supplementary material for: Genome and pan-genome analysis of a new exopolysaccharide-producing bacterium Pyschrobacillus sp. isolated from iron ores deposit and insights into iron uptake
Source: Front Microbiol. 2024 Aug 6;15:1440081. doi: 10.3389/fmicb.2024.1440081 (PMC11376405; doi:10.3389/fmicb.2024.1440081)
Supplement: Supplementary file 3 [file Table_3.DOCX]

## Table S3. Subsystem implicated in copper, cobalt, zinc, arsenic, cademium and chromium resistance based on RAST annotation of *Psychrobacillus* sp. NEAU-3TGS genome sequence

| **Subcategory** | **Subsystem** | **Feature (ID RASTannotation)** |
| --- | --- | --- |
| **Resistance to antibiotics and toxic compounds** | [Copper homeostasis](https://rast.nmpdr.org/seedviewer.cgi?page=Subsystems&subsystem=Copper_homeostasis&organism=1221880.7) | fig\|1221880.7.peg.483, [fig\|1221880.7.peg.1714](https://rast.nmpdr.org/seedviewer.cgi?page=Annotation&feature=fig\|1221880.7.peg.1714), [fig\|1221880.7.peg.4330](https://rast.nmpdr.org/seedviewer.cgi?page=Annotation&feature=fig\|1221880.7.peg.4330)  [fig\|1221880.7.peg.4203](https://rast.nmpdr.org/seedviewer.cgi?page=BrowseGenome&feature=fig\|1221880.7.peg.4203) |
|  | Cobalt-zinc- cadmium-resistance | fig\|1221880.7.peg.1116fig\|1221880.7.peg.2047fig\|1221880.7.peg.2372fig\|1221880.7.peg.2529fig\|1221880.7.peg.3086fig\|1221880.7.peg.3210 fig\|1221880.7.peg.3773fig\|1221880.7.peg.695 |
|  | [Zinc resistance](https://rast.nmpdr.org/seedviewer.cgi?page=Subsystems&subsystem=Zinc_resistance&organism=1221880.7) | fig\|1221880.7.peg.1512fig\|1221880.7.peg.3128 fig\|1221880.7.peg.570 |
|  | Arsenic resistance | [fig\|1221880.7.peg.301](https://rast.nmpdr.org/seedviewer.cgi?page=Annotation&feature=fig\|1221880.7.peg.301) [fig\|1221880.7.peg.302](https://rast.nmpdr.org/seedviewer.cgi?page=Annotation&feature=fig\|1221880.7.peg.302) [fig\|1221880.7.peg.3666](https://rast.nmpdr.org/seedviewer.cgi?page=Annotation&feature=fig\|1221880.7.peg.3666) [fig\|1221880.7.peg.3667](https://rast.nmpdr.org/seedviewer.cgi?page=Annotation&feature=fig\|1221880.7.peg.3667) [fig\|1221880.7.peg.4548](https://rast.nmpdr.org/seedviewer.cgi?page=Annotation&feature=fig\|1221880.7.peg.4548)  [fig\|1221880.7.peg.300](https://rast.nmpdr.org/seedviewer.cgi?page=Annotation&feature=fig\|1221880.7.peg.300)  fig\|1221880.7.peg.1034  fig\|1221880.7.peg.303 |
|  | Cadmium resistance | fig\|1221880.7.peg.482 |
|  | Chromium resistance | fig\|1221880.7.peg.1314 |
