# Supplementary material for: Genome and pan-genome analysis of a new exopolysaccharide-producing bacterium Pyschrobacillus sp. isolated from iron ores deposit and insights into iron uptake
Source: Front Microbiol. 2024 Aug 6;15:1440081. doi: 10.3389/fmicb.2024.1440081 (PMC11376405; doi:10.3389/fmicb.2024.1440081)
Supplement: Supplementary file 4 [file Table_4.DOCX]

**Table S4.** The functional Clusters of Orthologous Groups (COGs) of *Psychrobacillus* species (expressed in %)

| **Name** | ***P. vulpis*** | ***P. faecigallinarum*** | ***P. lasiicapitis***  ***NEAU-3TGS17*** | ***P. psychrodurans*** | ***P. psychrotolerans*** | ***P.***  ***soli*** | [***P. insolitus***](https://img.jgi.doe.gov/cgi-bin/m/main.cgi?section=TaxonDetail&page=taxonDetail&taxon_oid=2754412937) | [***P. glaciei***](https://img.jgi.doe.gov/cgi-bin/mer/main.cgi?section=TaxonDetail&page=taxonDetail&taxon_oid=2883947738) | ***Psychrobacillus* sp. NEAU-3TGS** | [***P. antarcticus***](https://www.ncbi.nlm.nih.gov/datasets/taxonomy/2879115) **Val9** |
| --- | --- | --- | --- | --- | --- | --- | --- | --- | --- | --- |
| E:Amino acid transport and metabolism | 9.78 | 10.68 | 11.66 | 9.33 | 10.13 | 12.10 | 9.50 | 9.85 | 9.54 | 10.28 |
| G: Carbohydrate transport and metabolism | 4.43 | 5.1 | 4.88 | 6.39 | 6.65 | 5.54 | 3.56 | 4.81 | 3.18 | 5.5 |
| D:Cell cycle control, cell division, chromosome partitioning | 1.55 | 1.03 | 1.35 | 1.51 | 1.49 | 1.31 | 1.78 | 1.46 | 1.10 | 1.4 |
| N: Cell motility | 2 | 2 | 2.08 | 2.38 | 2.42 | 1.94 | 2.26 | 2.27 | 0.90 | 2.1 |
| M: Cell wall/membrane/envelope biogenesis | 4.57 | 3.68 | 3.88 | 4.45 | 4.62 | 4.06 | 4.12 | 4.65 | 3.52 | 4.4 |
| B : Chromatin structure and dynamics | 0.03 | 0.01 | 0.02 | 0.03 | 0.04 | 0.05 | 0.04 | 0.03 | 0 | 0 |
| H: Coenzyme transport and metabolism | 4.49 | 4.23 | 4.30 | 5.02 | 5.05 | 4.50 | 6.61 | 4.50 | 2.32 | 3.1 |
| V: Defense mechanisms | 3.04 | 2.58 | 2.50 | 3.01 | 2.35 | 2.03 | 2.02 | 2.61 | 1.67 | 1.19 |
| C: Energy production and conversion | 4.17 | 4.017 | 4.34 | 4.48 | 4.55 | 4.84 | 5.27 | 3.79 | 4.31 | 4.5 |
| W : Extracellular structures | 0.13 | 0.32 | 0.33 | 0.23 | 0.21 | 0.34 | 0.24 | 0.39 | 0 | 0 |
| S: Function unknown | 7.53 | 7.02 | 7.12 | 7.16 | 7.11 | 6.73 | 6.42 | 6.90 | 22.97 | 23.16 |
| R: General function prediction only | 11.49 | 11 .23 | 11.73 | 9.13 | 8.85 | 11.30 | 9.27 | 10.74 | 0 | 0 |
| P: Inorganic ion transport and metabolism | 6.20 | 5.7 | 5.79 | 5.55 | 5.65 | 6.63 | 7.49 | 5.62 | 4.77 | 6.3 |
| U: Intracellular trafficking, secretion, and vesicular transport | 1.15 | 0.99 | 0.91 | 0.97 | 1 | 1.14 | 1.19 | 1.10 | 1.34 | 1.19 |
| I: Lipid transport and metabolism | 4.09 | 4.35 | 4.25 | 4.35 | 4.62 | 5.44 | 5.78 | 3.87 | 2.90 | 3.5 |
| X : Mobilome: prophages, transposons | 1.28 | 0.9 | 0.84 | 0.74 | 0.46 | 0.75 | 0.36 | 2.82 | 0 | 0 |
| F: Nucleotide transport and metabolism | 2.91 | 3.87 | 2.94 | 3.18 | 3.38 | 2.93 | 3.33 | 2.88 | 3 | 3.2 |
| O: Posttranslational modification, protein turnover, chaperones | 4.06 | 4 | 3.69 | 3.98 | 3.73 | 3.29 | 3.60 | 4.13 | 2.15 | 2.9 |
| L:Replication, recombination and repair | 3.50 | 3 | 3.57 | 3.95 | 3.84 | 3.12 | 4.48 | 4.21 | 3.90 | 6.02 |
| Q: Secondary metabolites biosynthesis, transport and catabolism | 2.62 | 2.82 | 2.62 | 2.11 | 2.27 | 2.71 | 2.97 | 2.33 | 0.90 | 1.7 |
| T:Signal transduction mechanisms | 5.37 | 6.08 | 6 | 6.16 | 6.08 | 5.47 | 4.95 | 5.83 | 3.35 | 5.5 |
| K: Transcription | 8.33 | 8.089 | 8.81 | 8.03 | 7.32 | 7.94 | 6.77 | 8.05 | 0.58 | 8.1 |
| J: Translation, ribosomal structure and biogenesis | 7.29 | 7.03 | 6.38 | 7.86 | 8.21 | 5.86 | 8 | 7.16 | 4.94 | 5.5 |
| Not in COG | 25.97 | 30 | 24.99 | 35.65 | 32.75 | 22.06 | 33,68 | 28.90 | 6.35 | 6.3 |
