# Supplementary material for: Genome and pan-genome analysis of a new exopolysaccharide-producing bacterium Pyschrobacillus sp. isolated from iron ores deposit and insights into iron uptake
Source: Front Microbiol. 2024 Aug 6;15:1440081. doi: 10.3389/fmicb.2024.1440081 (PMC11376405; doi:10.3389/fmicb.2024.1440081)
Supplement: Supplementary file 6 [file Table_6.DOC]

**Table S6**. Iron acquisition and metabolism of *Psychrobacillus* species based on RAST annotation.

|  |  | *Psychrobacillus sp. NEAU-3TGS* | *P. lasiicapitis* NEAU-3TGS17 | *P. soli* NHI-2 | *P. vulpis* Z8 | *P. glaciei* PB01 | *P. psychrotolerans* DSM 11706 | *P. insolitus* DSM 5 | *P. faecigallinarum* Sa2BUA9 | *P . psychrodurans* DSM 11713 | *P. antarcticus* |
| --- | --- | --- | --- | --- | --- | --- | --- | --- | --- | --- | --- |
| **Bacillibactin uptake** | Fe-bacillibactin uptake system (FeuA) | + | - | - | - | - | - | - | + | - | - |
|  | Fe-bacillibactin uptake system (FeuB) | + | - | - | - | - | - | - | + | - | - |
|  | F[Fe-bacillibactin uptake system FeuC](https://rast.nmpdr.org/seedviewer.cgi?page=FunctionalRole&role=Fe-bacillibactin uptake system FeuC&subsystem_name=Bacillibactin_Siderophore) | + | - | - | - | - | - | - | + | - | - |
|  | FeuD[Fe-bacillibactin uptake system FeuD](https://rast.nmpdr.org/seedviewer.cgi?page=FunctionalRole&role=Fe-bacillibactin uptake system FeuD&subsystem_name=Bacillibactin_Siderophore) | + | - | - | - | - | - | - | - | - | - |
|  | yuiI [Trilactone hydrolase](https://rast.nmpdr.org/seedviewer.cgi?page=FunctionalRole&role=Trilactone hydrolase %5Bbacillibactin%5D siderophore&subsystem_name=Bacillibactin_Siderophore) | + | - | - | - | - | - | - | - | - | + |
| **Anthrachelin** | Fe-ABC2 : Iron compound ABC uptake transporter permease protein | + | + | + | - |  | + | + | + | + | + |
|  | Fe-ABC3 : Iron compound ABC uptake transporter -ATP-binding protein | + | - |  | - | - | - | - | - | - | - |
|  | Fe-ABC1 : Iron compound ABC uptake transporter substrate-binding protein | + | + | - | + | - | + | + | + | + | + |
| **Membrane binding protein** | X-ABC 3 Uncharacterized iron compound ABC uptake transporter | + | + |  | + | + | + | + | + | + |  |
|  | X-ABC 1 | + | - | - | - | + | - | - | - | - | + |
|  | X-ABC 2 | + | - | - | - | + | - | - | - | - | + |
|  | Iron(3+)-hydroxamate-binding protein FhuD | + | - | - | - | - | - | - | - | - |  |
