# Supplementary figures and images for: Genome and pan-genome analysis of a new exopolysaccharide-producing bacterium Pyschrobacillus sp. isolated from iron ores deposit and insights into iron uptake
Source: Front Microbiol. 2024 Aug 6;15:1440081. doi: 10.3389/fmicb.2024.1440081 (PMC11376405; doi:10.3389/fmicb.2024.1440081)

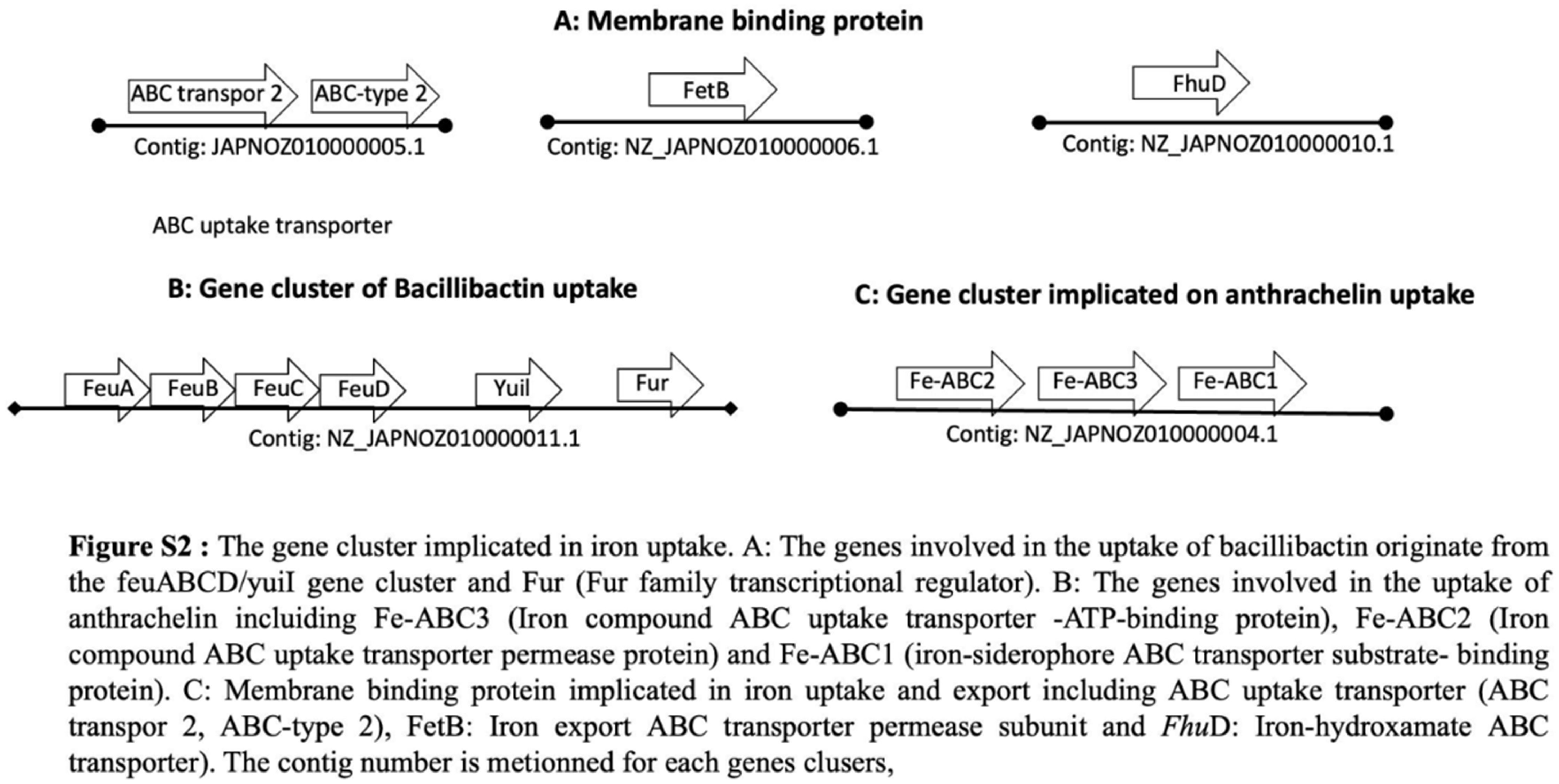

Supplement: Supplementary file 8 [file Image_2.TIFF]
